# Phenotypic plasticity, genetic structure and systematic position of *Neoechinorhynchus emyditoides* Fisher, 1960 (Acanthocephala: Neoechinorhynchidae): a parasite of emydid turtles from the Nearctic and Neotropical regions

**DOI:** 10.1017/S003118202200049X

**Published:** 2022-06

**Authors:** Ana Lucia Sereno-Uribe, Alejandra López-Jiménez, Marcelo Tonatiuh González-García, Carlos Daniel Pinacho-Pinacho, Rodrigo Macip Ríos, Martín García-Varela

**Affiliations:** 1 Departamento de Zoología, Instituto de Biología, Universidad Nacional Autónoma de México, Avenida Universidad 3000, Ciudad Universitaria, C.P. 04510, Ciudad de México, México; 2 Posgrado en Ciencias Biológicas, Universidad Nacional Autónoma de México, Avenida Universidad 3000, Ciudad Universitaria, C.P. 04510, Ciudad de México, México; 3 Investigador Cátedras CONACyT, Instituto de Ecología, A.C., Red de Estudios Moleculares Avanzados, Km 2.5 Ant. Carretera a Coatepec, Xalapa, Veracruz 91070, México; 4 Escuela Nacional de Estudios Superiores, Unidad Morelia, Universidad Nacional Autónoma de México, Edificio de Investigación y Posgrado, Antigua Carretera a Pátzcuaro No.8701, Col. Ex Hacienda de San José de la Huerta, C.P. 58190, Morelia, Michoacán, México

**Keywords:** Acanthocephalans, freshwater turtles, Gulf of Mexico, molecular markers, taxonomy

## Abstract

The taxonomy of the 10 recognized *Neoechinorhynchus* species associated with emydid turtles is complex due to the morphological conservatism. In the present study, specimens of *N. emyditoides* from northern and southeastern Mexico exhibit great phenotypic plasticity on its diagnostic characteristics. We sequenced three molecular markers: the internal transcribed spacers ITS1, ITS2 and 5.8S gene, the D2 + D3 domains of the large subunit from nuclear DNA and cytochrome *c* oxidase subunit I (*cox1*) from mitochondrial DNA. Sequences of the nuclear molecular markers were aligned and compared with other congeneric species associated with emydids available in GenBank. Phylogenetic analyses supported the polyphyly of *Neoechinorhynchus*. The species from emydids formed a clade, which was subdivided into five subclades that correspond with each species analysed (*N. pseudemydis*, *N. chrysemydis*, *N. emydis*, *N. schmidti* and *N. emyditoides*). To understand better the genetic structure of *N. emyditoides* a haplotype network was inferred with 29 *cox1* sequences, revealing the presence of 13 haplotypes, two of which were shared and 11 were unique. The high values of fixation index, *F*_st_ (0.4227–0.8925) detected between the two populations from southeastern and the two from northern Mexico indicated low genetic flow among the populations. Our data suggest that the *Neoechinorhynchus* species associated with emydid turtles diversified in the eastern USA and that of *N. emyditoides* expanded its distribution range reached southeastern Mexico.

## Graphical abstract

**Figure:**
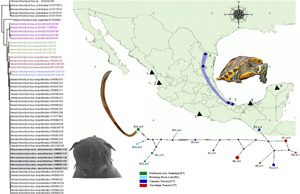

## Introduction

Phenotypic plasticity is defined as the ability of a genotype to produce multiple phenotypes in response to environmental conditions (Roff, 2002; Miner *et al*., 2005). In organisms with complex and diverse life cycles such as parasites, different environmental conditions include (1) the host's immune system, (2) different host species and (3) the geographical distribution of the definitive hosts (Poulin, 2007). The recent application of molecular markers has helped to define, recognize, delineate and better understand intraspecific variation that can be attributed to differences in the development and phenotypic plasticity of acanthocephalans (Steinauer *et al*., 2007; Rosas-Valdez *et al*., 2012, 2020; Alcántar-Escalera *et al*., 2013; Perrot-Minnot *et al*., 2018; Pinacho-Pinacho *et al*., 2018*a*; Lisitsyna *et al*., 2019).

The Neoechinorhynchidae (Ward, 1917) Van Cleave, 1928 is a large, globally distributed family of acanthocephalans, which are typically parasites from the intestine of teleost fishes and freshwater turtles. At present, the family includes 14 genera split into four subfamilies (Gibson and Wayland, 2022). The type genus *Neoechinorhynchus* Stiles and Hassall, 1905 represents a hyperdiverse group of endoparasites of marine, freshwater, brackish water fishes and freshwater turtles, with ~117 species distributed worldwide (Amin, 2013; Smales, 2013; Pinacho-Pinacho *et al*., 2018*a*; Amin *et al*., 2019, 2020). In the Americas, 50 species have been described: 33 in North America, corresponding to the Nearctic biogeographical region, and 17 in Middle and South America, corresponding to the Neotropical biogeographical region (Amin, 2013; Pinacho-Pinacho *et al*., 2018*b*). Members of *Neoechinorhynchus* have been the target of numerous studies related to their ecology, host–parasite relationships, pathogenicity, taxonomy and systematics (see references in Kennedy, 2006; Pinacho-Pinacho *et al*., 2012, 2014, 2018*a*; Amin, 2013; Smales, 2013; Amin *et al*., 2020; Koch *et al*., 2021).

The first morphological and molecular phylogenies inferred with a few species of *Neoechinorhynchus* revealed that the genus is paraphyletic (Pinacho-Pinacho *et al*., 2017). However, one of the most emblematic and enigmatic groups of the genus *Neoechinorhynchus* from North America associated with emydid turtles (Barger, 2004; Barger and Nickol, 2004) formed a monophyletic assemblage (García-Varela and Pinacho-Pinacho, 2018; Koch *et al*., 2021). The taxonomy of the 10 currently described species of *Neoechinorhynchus* associated with emydid turtles is complex due to their morphological homogeneity. Species delimitation relies entirely on characteristics of the female worm, including the contour of the posterior end and the shape and membrane structure of the fully formed egg, whereas male worms are nearly identical among species (Cable and Hopp, 1954; Barger, 2004; Barger and Nickol, 2004). In addition, Dezfuli and Tinti (1998) and Barger and Nickol (2004) mentioned that species of *Neoechinorhynchus* from turtles exhibit great phenotypic plasticity, which may lead to the misclassification of female specimens and leave males of different species completely indistinguishable. Barger (2004) performed one of the most comprehensive taxonomic reviews of *Neoechinorhynchus* species that infect emydid turtles from North America, acknowledging that eastern USA represents a biodiversity hot spot for this group of acanthocephalans. Barger reported that *Neoechinorhynchus emyditoides* Fisher, 1960 has the widest distribution range extending from the eastern USA to southeastern Mexico through the Gulf of Mexico.

As part of our long-term studies on the biodiversity of helminth parasites of turtles, acanthocephalans belonging to the Neoechinorhynchidae were recovered from the intestines of emydid turtles from five localities in northern and southeastern Mexico. Our extensive sampling allowed us recognize morphologically two species of acanthocephalans, i.e. *Neoechinorhynchus schmidti* Barger, Thatcher and Nickol, 2004 and *N. emyditoides*.

The objective of the current study was to combine morphological and molecular characteristics to investigate the phenotypic plasticity of *N. emyditoides* along its distribution range and explore the genetic structure of populations by using sequences of the cytochrome c oxidase subunit 1 (*cox1*) from mitochondrial DNA. In addition, we briefly discuss the systematics of *Neoechinorhynchus* species associated with emydid turtles by using sequences of two molecular markers: the D2 + D3 domain of the large subunit (LSU) and the internal transcribed spacer region, including 5.8S (ITS) from nuclear ribosomal DNA.

## Materials and methods

### Specimen collection

**Fig. 1.**
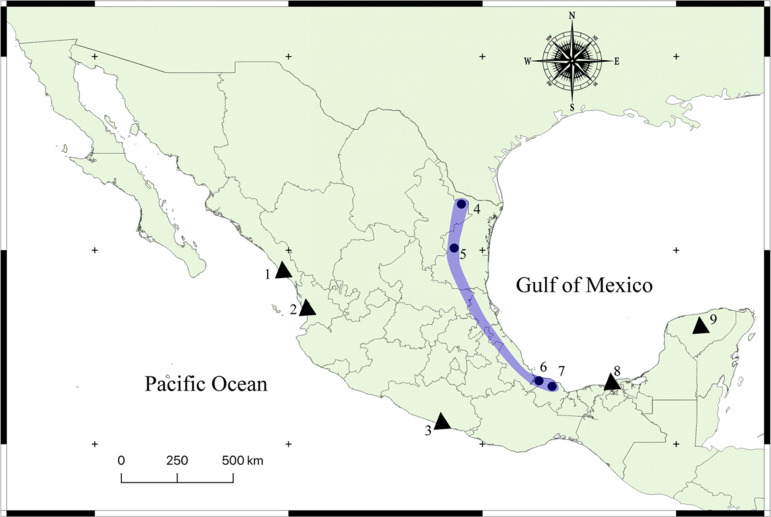
Map of Mexico showing the sampled sites for the turtles. Localities with a circle were positive for the infection with *Neoechinorhynchus emyditoides.* Localities with a triangle were negative for the infection. Localities: (1) Huizache, Sinaloa; (2) La Tovara, Nayarit; (3) Tres Palos, Guerrero; (4) Monterrey, Nuevo Leon; (5) Purificación river, Tamaulipas; (6) Tlacotalpan, Veracruz; (7) Catemaco, Veracruz; (8) Pantanos de Centla, Tabasco; (9) Holcá, Yucatán.

A total of 47 turtles including 28 specimens of Meso-American slider [*Trachemys scripta venusta* (Gray)], 6 specimens of ornate slider [*Trachemys ornata* (Gray)], 2 of red-eared slider [*Trachemys scripta elegans* (Schoepff)], 4 of yellow mud turtle [*Kinosternon flavescens* (Agassiz)], plus 7 specimens of Mexican musk turtle [*Staurotypus triporcatus* (Wiegmann)] were collected from previous field expedition [see Pinacho-Pinacho *et al*. (2014, 2018*a*) and García-Varela and Pinacho-Pinacho (2018) in 9 localities across Mexico (Fig. 1; Table S1)]. Turtles were dissected within 2 h after capture; their viscera were placed in separate Petri dishes containing a 0.75% saline solution and examined under a dissecting microscope. The turtles identified as *T. scripta venusta* were positive for the infection with acanthocephalans, which were washed in 0.75% saline solution and placed in distilled water at 4°C overnight and subsequently preserved in 100% ethanol. Turtles were identified using the field guide of Legler and Vogt (2013).

### Morphological study

For taxonomic identification, specimens were stained with Mayer's paracarmine, dehydrated in a graded ethanol series, cleared with methyl salicylate and mounted on permanent slides with Canada balsam. Mounted specimens were examined under a bright-field Leica DM 1000 LED microscope (Leica, Wetzlar, Germany), and drawings were made using a drawing tube attached to the microscope. Measurements were taken using Leica Application Suite microscope software (Leica) and are given in micrometres (*μ*m).

Vouchers of *N. emyditoides* from Tlacotalpan, Veracruz (No. 6695) and the new vouchers (Nos. 11647–11653) were examined and deposited in the Colección Nacional de Helmintos (CNHE), Instituto de Biología, Universidad Nacional Autónoma de México (UNAM), Ciudad de México, Mexico. The species identification was conducted following the revision of the genus *Neoechinorhynchus* by Barger (2004) and the original description (Fisher, 1960). For scanning electron microscopy (SEM), two specimens of *N. emyditoides* of each locality sampled were dehydrated with an ethanol series, critical point dried, sputter coated with gold and examined with a Hitachi Stereoscan Model S-2469N scanning electron microscope operating at 15 kV from the Instituto de Biología, UNAM.

### Amplification and sequencing of DNA

A total of seven specimens identified as *N. emyditoides* were analysed. The posterior end of four specimens, two male and two female (hologenophores; Pleijel *et al*., 2008), were used for DNA extraction, whereas the rest of the body was stained with Mayer's paracarmine and mounted on permanent slides with Canada balsam. In addition, one female of *N. emyditoides* from Purification River was cut-off into three sections. The anterior and posterior sections were processed for SEM, whereas the middle section was processed for DNA extraction. Finally, other two specimens identified as *N. emyditoides* were placed individually in tubes and digested overnight at 56°C in a solution containing 10 mm Tris–HCl (pH 7.6), 20 mm NaCl, 100 mm Na_2_ EDTA (pH 8.0), 1% sarkosyl and 0.1 mg mL^−1^ proteinase K. Following digestion, DNA was extracted from the supernatant using the DNAzol reagent (Molecular Research Center, Cincinnati, OH, USA) according to the manufacturer's instructions. The domains D2 + D3 and ITS from nuclear ribosomal DNA and the *cox1* from mitochondrial DNA were amplified using polymerase chain reaction (PCR). The domains D2 + D3 from LSU of approximately 700 bp were amplified using forward primer 502, 5′-CAA GTA CCG TGA GGG AAA GTT GC-3′ and reverse primer 536, 5′-CAG CTA TCC TGA GGG AAAC-3′ (García-Varela and Nadler, 2005). A fragment of approximately 750 bp from ITS was amplified using the forward primer BD1, 5′-GTC GTA ACA AGG TTT CCG TA-3′ and the reverse primer BD2, 5′-ATC TAG ACC GGA CTA GGC TGT G-3′ (Luton *et al*., 1992). The *cox1* of approximately 550 bp was amplified using the forward primer 516, 5′-ATT TTT TAG TTT GAG TGT GAG GAG-3′ and the reverse primer 517, 5′-ATGA CGA ATT AAT ATT ACG ATC CA-3′ (see Pinacho-Pinacho *et al*., 2018*a*, 2018*b*). The amplification reactions (25 *μ*L) consisted of 1 *μ*L of each primer (10 *μ*m), 2.5 *μ*L of 10× buffer, 1.5 *μ*L of 2 mm MgCl_2_, 0.5 *μ*L of dNTPs (10 mm), 16.37 *μ*L of water, 2 *μ*L of genomic DNA and 1 U of Taq DNA polymerase (Platinum Taq, Invitrogen Corporation, São Paulo, Brazil). The PCR cycling conditions for amplification included denaturation at 94°C for 3 min, followed by 35 cycles of 94°C for 1 min, annealing at 50°C for LSU, ITS and 40°C for *cox1* for 1 min and extension at 72°C for 1 min, with a final postamplification incubation at 72°C for 10 min. The sequencing reactions were performed using the initial primers for LSU, ITS and *cox1*, plus two internal primers: 503, 5′-CCT TGG TCC GTG TTT CAA GAC G-3′ and 504, 5′-CGT CTT GAA ACA CGG ACT AAGG-3′ for LSU (García-Varela and Nadler, 2005). Sequencing reactions were performed using ABI Big Dye (Applied Biosystems, Boston, MA, USA) terminator sequencing chemistry, and the reaction products were separated and detected using an ABI 3730 capillary DNA sequencer. Contigs were assembled, and base-calling differences were resolved using CodonCode Aligner 9.0.1 (CodonCode Corporation, Dedham, MA, USA). Newly generated sequences were deposited in the GenBank database under the accession numbers; Hologenophore, male (OM892136 and OM892138 for LSU; OM892142 and OM892144 for ITS; OM891889 for *cox1*), female (OM892137 and OM892139 for LSU; OM892143 and OM892145 for ITS; OM891888 and OM891890 for *cox1*). The accession numbers for the female of *N. emyditoides* cut-off into three sections were OM892146 for ITS and OM892140 for LSU. Finally, the accession numbers of the other specimens were OM892141 for LSU and OM892147-148 for ITS.

### Alignments and phylogenetic analyses

Sequences obtained in the current research for LSU and ITS were aligned with other congeneric species downloaded from GenBank (see Table 1). In addition, sequences of the LSU and ITS of other genera, such as *Polyacanthorhynchus* Travassos 1920, *Acanthosentis* Verma and Datta 1929, *Floridosentis* Ward 1953, *Mayarhynchus* Pinacho-Pinacho, Hernández-Orts, Sereno-Uribe, Pérez-Ponce de León and García-Varela, 2017, and *Atactorhynchus* Chandler 1935, were used as outgroup. Sequences of each nuclear molecular marker were aligned separately using the software Clustal W with default parameters implemented in MEGA version 7.0 (Kumar *et al*., 2016). The best-fitting nucleotide substitution models for LSU and ITS dataset were GTR + G + I and were estimated with the Akaike Information Criterion (AIC) implemented in MEGA version 7.0 (Kumar *et al*., 2016). The phylogenetic analyses were inferred through maximum likelihood (ML) with the program RAxML v7.0.4 (Silvestro and Michalak, 2012) and Bayesian inference (BI) criteria, employing the nucleotide substitution model identified for AIC. BI trees were generated using MrBayes v3.2 (Ronquist *et al*., 2012), running two independent MC3 runs of four chains for 5 million generations and sampling tree topologies every 1000 generations. ‘Burn-in’ periods were set to 1 million of generations according to the standard deviation of split frequencies values (<0.01). To support each node, 10 000 bootstrap replicates were run with the ML method. Posterior probabilities of clades were obtained from 50% majority rule consensus of sample trees after excluding the initial 20% as ‘burn-in’. The genetic divergence among taxa was estimated using uncorrected ‘p’ distances with the program MEGA version 7.0 (Kumar *et al*., 2016).

**Table 1.** Specimens information, GenBank accession numbers of LSU, ITS and *cox* 1

			GenBank			
	Host	Locality	LSU	*COX* 1	ITS	References
***Neoechinorhynchus*** Stiles and Hassall, 1905						
*N. emyditoides* Fisher, 1960	*Trachemys scripta venusta*	Catemaco, Veracruz, Mexico	HQ634781; KR086315; KR089315–321	KY077094–095	KY077108–109 MG870838–845	Pinacho-Pinacho *et al*. (2018*a*, 2018*b*)
	*Trachemys scripta venusta*	Purificación river, Tamaulipas, Mexico	KY077082; **OM892136** **OM892137** **OM892138** **OM892139** **OM892140** **OM892141**	MK089514 **OM891888** **OM891889** **OM891890**	MK089807 **OM892142** **OM892143** **OM892144** **OM892145** **OM892146** **OM892147** **OM892148**	Pinacho-Pinacho *et al*. (2018*a*, 2018*b*); García-Varela and Pinacho-Pinacho (2018). **This study**
	*Trachemys scripta venusta*	Tlacotalpan, Veracruz, Mexico	KR086322–328		MG870846–854	Pinacho-Pinacho *et al*. (2014)
	*Trachemys scripta elegans*	Monterrey, Nuevo Leon, Mexico	KR086329–334		MG870855–863	Pinacho-Pinacho *et al*. (2014, 2018*a*, 2018*b*)
	*Trachemys scripta elegans*	Oklahoma, Texas, USA	MK238158		MW520495–497	Reyda and Phillips 2019 (unpublished); Koch *et al*. (2021)
*N. pseudemydis* Cable and Hopp, 1954	*Trachemys scripta elegans*	Oklahoma, USA	MK238159–160		MW520486–490; MK238090–091	Reyda and Phillips 2019 (unpublished); Koch *et al*. (2021)
*N. chrysemydis* Cable and Hopp, 1954	*Trachemys scripta elegans*	Oklahoma, USA	MK238161–162		MK238092–093; MW520491 MW520493–494	Reyda and Phillips 2019 (unpublished); Koch *et al*. (2021)
*N. emydis* (Leidy, 1851) (Van Cleave, 1916)	*Trachemys scripta elegans*	Oklahoma, USA	MK238154–157		MW520481–484	Reyda and Phillips 2019 (unpublished); Koch *et al*. (2021)
*N. schmidti* Barger, Thatcher and Nickol, 2004	*Trachemys scripta*	Centla, Tabasco, Mexico	HQ634785–788; FJ389001		KC004172–173; MG870835–837	García-Varela *et al*. (2011); Pinacho-Pinacho *et al*. (2018*a*, 2018*b*)
*Neoechinorhynchus* sp.	*Physocypria* sp.	Oklahoma, USA			MW520470–480	Koch *et al*. (2021)
*Neoechinorhynchus* sp.	*Planorbella cf. trivolvis*	Oklahoma, USA			MW520457–469	Koch *et al*. (2021)
*N. roseum* Salgado-Maldonado, 1978	*Citharichthys gilberti* Jenkins and Evermann	La Tovara, Nayarit, Mexico	FJ389000	JN830868	FJ388981	Martínez-Aquino *et al*. (2009)
	*Achirus mazatlanus* (Steindachner)	Caimanero Lagoon, Sinaloa, Mexico	FJ388999	JN830867	FJ388980	Martínez-Aquino *et al*. (2009)
*N. panucensis* Salgado-Maldonado, 2013	*Herichthys cyanoguttatus* Baird and Girard	River Purificacion, Tamaulipas, Mexico	KY077070–KY077072	MK089511–513	MK089804	Pinacho-Pinacho *et al*. (2018*a*, 2018*b*); García-Varela and Pinacho-Pinacho (2018)
	*Herichthys* sp.	River Pantepec, Veracruz, Mexico	KR086335	KY077087	KY077103	Pinacho-Pinacho *et al*. (2015); Pinacho-Pinacho *et al*. (2018*a*, 2018*b*)
*N. golvani* Salgado-Maldonado, 1978	*Paratheraps fenestratus* (Günther)	Lake Catemaco, Veracruz, Mexico	FJ388986; KR086272	KY077088; JN830852	FJ388967; KC004224	Martínez-Aquino *et al*. (2009)
*N. costarricense*	*Parachromis managuensis*	Lake Jalapa, Costa Rica	KR086239–240		MG870741–742	Pinacho-Pinacho *et al*. (2018*a*, 2018*b*)
*N. chimalapasensis* Salgado-Maldonado, Caspeta-Mandujano and Martínez-Ramírez, 2010	*Awaous banana* (Valenciennes)	River Negro, Santa Maria Chimalapa, Oaxaca, Mexico	KR086336–337	KY077089–090	KY077104–105	Pinacho-Pinacho *et al*. (2014); Pinacho-Pinacho *et al*. (2018*a*, 2018*b*)
*N. brentnickoli* Monks, Pulido-Flores and Violante-González, 2011	*Dormitator latifrons* (Richardson)	Tres Palos Lagoon, Guerrero, Mexico	FJ388991; KR086219	KY077091; JN830808	FJ388972; KC004184	Martínez-Aquino *et al*. (2009)
*N. mexicoensis* Pinacho-Pinacho, Sereno-Uribe and García-Varela, 2014	*Dormitator maculatus* (Bloch)	River Papaloapan, Tlacotalpan, Veracruz, Mexico	KR086299; KR086301	KY077092–093	KY077106–107	Pinacho-Pinacho *et al*. (2014)
*N. mamesi* Pinacho-Pinacho, Pérez-Ponce de León and García-Varela, 2012	*Dormitator latifrons* (Richardson)	Joaquin Amaro Lagoon, Chiapas, México	JN830772; JN830774	JN830799–800	KC004193; KC004191	Pinacho-Pinacho *et al*. (2012)
*N. saginatus* Van Cleave and Bangham, 1949	–	–	AY829091	DQ089704	FJ388984	García-Varela and Nadler (2005)
*N. cylindratus* (Van Cleave, 1914) Van Cleave, 1919	*Micropterus salmoides* (Lacepède)	River Purificacion, Tamaulipas, Mexico	KY077073–KY077076	MK089515–517	MK089806–807 MK089918–919	Pinacho-Pinacho *et al*. (2018*a*, 2018*b*); García-Varela and Pinacho-Pinacho (2018)
*N. salmonis* Ching, 1984	*Salvelinus malma* (Walbaum)	Lake Chistoe	–	KF156889	–	Malyarchuk *et al*. (2014)
*N. tumidus* Van Cleave and Bangham, 1949	*Salvelinus alpinus* (Linnaeus)	Lake L'distoe; Lake Engteri, Russia	–	KF156887; KF156886	–	Malyarchuk *et al*. (2014)
*N. beringianus* Mikhailova and Atrashkevich, 2008	*Pungitius pungitius* (Linnaeus)	Lake Chernoe; Grand Lake, Russia	–	KF156882	–	Malyarchuk *et al*. (2014)
*N. simansularis* Roitman, 1961	*Salvelinus alpinus* (Linnaeus)	Lake Engteri, Russia	–	KF156890	–	Malyarchuk *et al*. (2014)
*Neoechinorhynchus* sp.	*Coregonus nasus* (Pallas); *Prosopium cylindraceum* (Pennant); *Coregonus lavaretus* (Linnaeus)	River Kolyma; Lake Rybnoe; River Yana, Russia	–	KF156884; KF156888; KF156885; KF156883	–	Malyarchuk *et al*. (2014)
*Neoechinorhynchus* sp.	–	–	KY077077–078	–	KY077110–112	Pinacho-Pinacho *et al*. (2018*a*, 2018*b*)
*Neoechinorhynchus* sp.	–	–	HQ634789	–		García-Varela *et al*. (2011)
***Floridosentis*** Ward 1953						
*F. mugilis* (Machado, 1951)	*Mugil cephalus* Linnaeus	Sontecomapan Lagoon, Veracruz, Mexico	JQ436497; JQ436495	DQ089723	KC004178–179	Rosas-Valdez *et al*. (2012)
*F. pacifica* Bravo-Hollis, 1969	*Mugil curema* Valenciennes	Tres Palos Lagoon, Guerrero, Mexico	JQ436531; JQ436533	–	–	Rosas-Valdez *et al*. (2012)
***Mayarhynchus*** Pinacho-Pinacho, Hernández-Orts, Sereno-Uribe, Pérez-Ponce de León and García-Varela, 2017						
*M. karlae* Pinacho-Pinacho, Hernández-Orts, Sereno-Uribe, Pérez-Ponce de León and García-Varela, 2017	*Thorichthys ellioti* Meek	Tlacotalpan, Veracruz, Mexico	KY077066–KY077068	KY077083–KY077085	KY077098–KY077100	Pinacho-Pinacho *et al*. (2018*a*, 2018*b*)
	*Paratheraps fenestratus* (Günther)	Tlacotalpan, Veracruz, Mexico	–	KY077086	KY077101	Pinacho-Pinacho *et al*. (2018*a*, 2018*b*)
	*Mayaheros urophthalmus* (Günther)	Silvituc Lagoon, Campeche, Mexico	KY077069	–	KY077102	Pinacho-Pinacho *et al*. (2018*a*, 2018*b*)
*Octospiniferoides* sp.^a^	*Petenia splendida* (Günther)	River Champoton, Campeche, Mexico	FJ388997	–	FJ388978	Unpublished
***Polyacanthorhynchus*** Travassos, 1920 *P. caballeroi* Diaz-Ungria and Rodrigo, 1960	*Caiman yacare* Daudin	Bolivia	DQ089738	DQ089724		García-Varela and Nadler (2005)
***Atactorhynchus*** Chandler, 1935 *A. duranguensis* Salgado-Maldonado, Aguilar-Aguilar and Cabañas-Carranza, 2005	*Cyprinodon meeki* Miller	Durango, Mexico	KY077079–KY077081	KY077096–097	KY077113–KY077115	Pinacho-Pinacho *et al*. (2018*a*, 2018*b*)
***Acanthosentis*** *A. cheni*	*Coilia nasus*	Yangtze River		JX960752		Song *et al*. (2014)

Sequences in bold were generated in the current study.

a Source for sequences misidentified as *Octospiniferoides* sp.

To examine *cox1* haplotype frequency among the populations of *N. emyditoides*, an unrooted statistical network was constructed using the PopART program with the median-joining algorithm (Bandelt *et al*., 1999). The degree of genetic differentiation among the populations was estimated using the fixation indices *F*_st_ (Hudson *et al*., 1992), with the program Arlequin v.3.5 (Excoffier and Lischer, 2010). To investigate the population history and demography, Tajima's *D* (Tajima, 1989) and Fu's *F* (Fu, 1997) tests were calculated using DnaSP v. 5. 10 (Rozas *et al*., 2003). Values less than 0.05 were considered statistically significant.

## Results

### Morphological identification

**Fig. 2.**
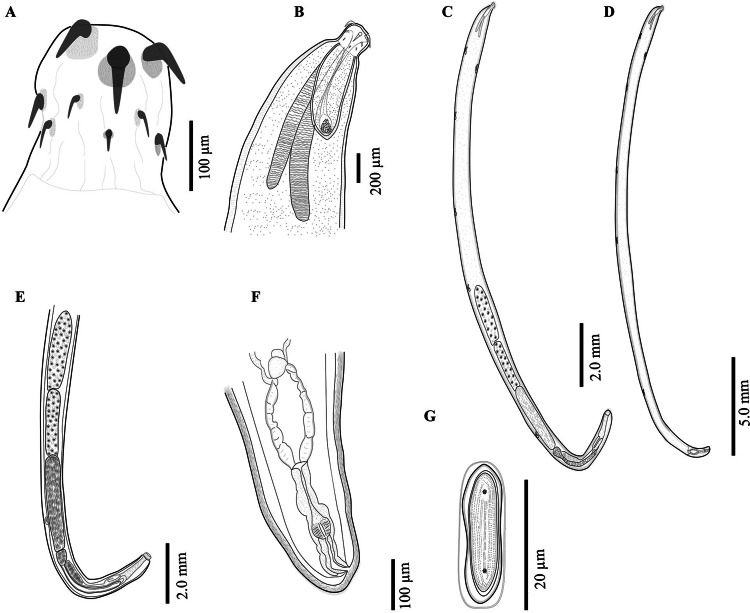
*Neoechinorhynchus emyditoides* from *Trachemys scripta venusta* from Catemaco Veracruz, Mexico. Proboscis (A); male anterior region (B); adult male, whole worm (C); adult female, whole worm (D); male reproductive system (E); female reproductive system (F); egg (G).

**Fig. 3.**
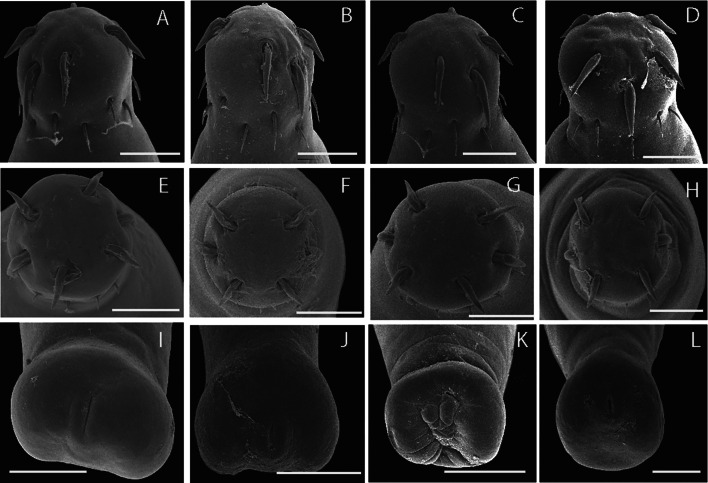
Scanning electron micrographs of proboscis (A–H) and anterior region of adult female (I–L) of *Neoechinorhynchus emyditoides* of the four populations sampled; Purificación river, Tamaulipas (A, E, I) from *Trachemys scripta venusta*; Tlacotalpan, Veracruz (B, F, J) from *Trachemys scripta venusta*; Catemaco, Veracruz (C, G, K) from *Trachemys scripta venusta*; Monterrey, Nuevo Leon (D, H, L) from *Trachemys scripta elegans*. Scale bars = 100 *μ*m.

The acanthocephalans were collected from several previous field expeditions in four localities (Fig. 1; Table 1), two from the northern Mexico and two from the southeastern Mexico (Figs 2 and 3). The acanthocephalans exhibited the typical morphological characteristics of *N. emyditoides.* For example, trunk elongated cylindrical, slender, curved ventrally, body wall with reticular lacunar system, usually five (occasionally three) dorsal giant hypodermic nuclei and one ventral (Fig. 2C–D). Proboscis nearly cylindrical, longer than wide (Figs 2A and 3A–D). Anterior hooks markedly larger than middle hooks not in a perfect circle but at different levels, posterior hooks smaller than middle hooks (Figs 2A and 3A–H). Neck short, wider than longer. Lemnisci appreciably different in length; longer than the proboscis receptacle. Proboscis receptacle single-walled (Fig. 2B). Male with two oblong testes, contiguous or slightly overlapping, equatorial, just behind anterior trunk; anterior testis relatively longer than the posterior testis. Prominent cement gland elongated, longer than anterior testis. Rounded-ovoid cement gland reservoir with two lateral ducts. Saefftingen's pouch beginning at level of anterior end of the cement reservoir duct (Fig. 2E). Posterior end of the female usually rounded with two lobes (Figs 2F and 3I–L), short vagina, slim uterus and uterine bell attached to the anterior body wall (Fig. 2F). Elliptical eggs, slightly inflated at the poles (Fig. 2G). These characteristics of our new morphometric data correspond to those reported previously (see Table 2) (Bravo-Hollis, 1946; Fisher, 1960; Barger, 2004).

**Table 2.** Comparative measurements of specimens of *Neoechinorhynchus emyditoides*

Characters host	Fisher, 1960 Arkansas, USA *T. scripta elegans*	Bravo-Hollis, 1946 Tlacotalpan, Veracruz *T. scripta venusta*	García-Varela *et al*., 2011 Tlacotalpan, Veracruz *T. scripta venusta*	This study Purificación, river Tamaulipas *T. scripta venusta*	This study Catemaco, Veracruz *T. scripta venusta*	This study Monterrey, Nuevo León. *T. scripta elegans*
*Male*		*N* = 6	*N* = 7	*N* = 5	*N* = 8	*N* = 6*
Trunk *L* × *W* (mean) (mm)	23.1 × 842	10.7–18.4 × 569–600	9.0–22.4 (16.1) × 174–833 (513)	10–16.2 (13.7) × 404–693 (576)	15–22.7 (19.8) × 593–1031 (898)	9.1–12.2(10.6) × 394–712 (579)
Proboscis *L* × *W* (mean) (mm)	–	174–205 × 166–190	133–186 (158) × 101–163 (141)	166–268 (216) × 155–220 (192)	175–229 (205) × 160–202 (191)	172–223 (579) × 150–204 (194)
Receptacle *L* × *W* (mean)	420–750 × 152–203	395–474 × 205–237	386–494 (439) × 151	438–599 (532) × 156–255 (206)	496–569 (550) × 174–245 (215)	387–554 (490) × 171–230 (207)
Hooks *L* (mean)						
Lateral of anterior circle	78–108	–	58–83 (67)	66–81 (74)	70–76 (74)	63–78 (75)
No. 1	71–100	75–84	57–77 (65)	87–100 (93)	80–96 (92)	77–102 (98)
No. 2	45–61	45–52	35–41 (37)	42–65 (55)	40–50 (46)	36–50 (48)
No. 3	22–58	14–17	18–34 (25)	20–34 (29)	25–31 (28)	20–33 (28)
Longer lemiscus *L* (mean)	–	711–1090	840–1201 (1061)	906–1037 (978)	1088–1606 (1320)	1051–1316 (1232)
Shorter lemiscus *L* (mean)	–	699–806	805–1066 (967)	871–989 (932)	920–1331 (1200)	856–1255 (1083)
Anterior testis *L* × *W* (mean)	1386–1504 × 380–472	790–1427 × 229–269	535–1958 (1385) × 168–527 (313)	980–1459 (1232) × 290–527 (390)	1726–2646 (2343) × 296–651 (533)	780–957 (868) × 243–274 (258)
Posterior testis *L* × *W* (mean)	1118–1386 × 311–483	474–995 × 221–269	743–2323 (1368) × 216–494 (340)	708–1410 (1110) × 272–467 (396)	1184–2228 (2133) × 317–655 (485)	677–1039 (858) × 217–239 (228)
Cement gland *L* × *W* (mean)	1450–1800 × 248–300	-	991–2844 (1779) × 155–394 (266)	1131–2025 (1693) × 291–382 (340)	1418–2960 (2470) × 264–480 (402)	576–1397 (851) × 168–224 (198)
Cement reservoir *L* × *W* (mean)	–	332–533 × 221–300	251–572 (473) × 131–346 (286)	346–385 (365) × 288–309 (298)	390–563 (488) × 316–341 (338)	147–306 (246) × 127–174 (158)
Cement reservoir duct *L* (mean)	–		1281–1500 (1389)	1283–1506 (1394)	1264–1792 (1566)	1036–1542 (1407)
*Females*		*N* = 10	*N* = 7	*N* = 7	*N* = 2	*N* = 4*
Trunk *L* × *W* (mean) (mm)	34.3 × 940	13.4–31.2 × 506–743	13.7–32.6 (22.9) × 378–756 (557)	15–19.2 (17.6) × 427–765 (583)	22–25 (23.6) × 690–787 (738)	6.9–13.4 (9.5) × 395–590 (473)
Proboscis *L* × *W* (mean) (mm)	–	205–237 × 158–190	156–213 (181) × 107–208 (140)	183–257 (220) × 173–212 (190)	221–232 (226) × 176–193 (184)	180–228 (206) × 156–204 (181)
Hooks *L* (mean)						
Lateral of anterior circle	78–108	–	54–75 (60)	63–89 (74)	63–72 (67.5)	72–94 (80)
No. 1	71–100	77–94	76–83 (79)	85–106 (98)	79–89 (84)	89–101 (96)
No. 2	45–61	49–52	38–43 (40)	40–52 (45)	41–44 (42)	34–47 (40)
No. 3	22–58	28–45	18–32 (22)	20–34 (25)	22–25 (23.5)	25–32 (30)
Longer lemiscus *L* (mean)		711–948	683–1045 (864)	908–1358 (1136)	1101–1459 (1280)	1095–1418 (1256)
Shorter lemiscus *L* (mean)	–	711	–	1056–1433 (1291)	1047–1157 (1102)	1025–1182 (1103)
Reproductive system (from mouth of uterine bell to genital pore) *L* (mean) (mm)	–	402–667	317–527 (414)	468–587 (524)	438–472 (455)	415–473 (444)
Uterus *L* (mean) (mm)		182–284	181–309 (262)	222–307 (268)	202–385 (293)	228–278 (253)
Vagina *L* (mean) (mm)	0.135–0.203	115–178	86–104 (97.5)	61–75 (69)	64–75 (69.5)	61–81 (71)
Eggs *L* × *W* (mean) (mm)	25–30 × 10–13	18–20 × 7–8	16–18 (16.5) × 6–8 (7)	25–28 (22) × 10–12 (11)	24–27 (25) × 9–11 (10)	–

*N*, number of specimens used; *, immature specimens.

### Remarks

Fisher (1960) recognized two previous described species [*Neoechinorhynchus pseudemydis* Cable and Hopp, 1954 and *Neoechinorhynchus emydis* (Leidy, 1851) Van Cleave, 1919] and described the species *N. emyditoides* from diverse definitive hosts: *Trachemys scripta scripta*, *T. scripta elegans*, *T. ornata* and *Emydoidea blandingii* (Holbrook), from Arkansas, Texas, Virginia, Massachusetts, California zoo in the USA, and southeastern Mexico (Bravo-Hollis, 1946; Fisher, 1960). Barger (2004) and Barger and Nickol (2004) performed one of the most comprehensive taxonomic reviews of the species of *Neoechinorhynchus* that infects emydid turtles, mentioning that *N. emyditoides* is the species with the most extensive distribution range including 11 states from eastern USA and single state (Veracruz) from southeastern Mexico. In the current research, we analysed the morphology of specimens from four populations of *N. emyditoides* from Mexico, exhibiting a wide phenotypic plasticity along its distribution. For instance, newly collected material showed some level of morphological intraspecific variation with respect to previous descriptions performed by Fisher (1960), Bravo-Hollis (1946) and García-Varela *et al*. (2011) (see Table 2). Interestingly, the specimens from Mexico possess lower limits for the following characteristics: body size, receptacle proboscis and proboscis hooks length with respect to original description (see Table 2). Cable and Hopp (1954) mentioned that the size and shape of fully developed eggs and the posterior end of the female are morphological traits keys on the differentiation at species level. For instance, the female of *N. emyditoides* is characterized by possessing a posterior end rounded with two lobes. Our specimens showed phenotypic plasticity on the shape of the posterior end (see Fig. 3I–L) (see Barger, 2004).

### Phylogenetic analyses

**Fig. 4.**
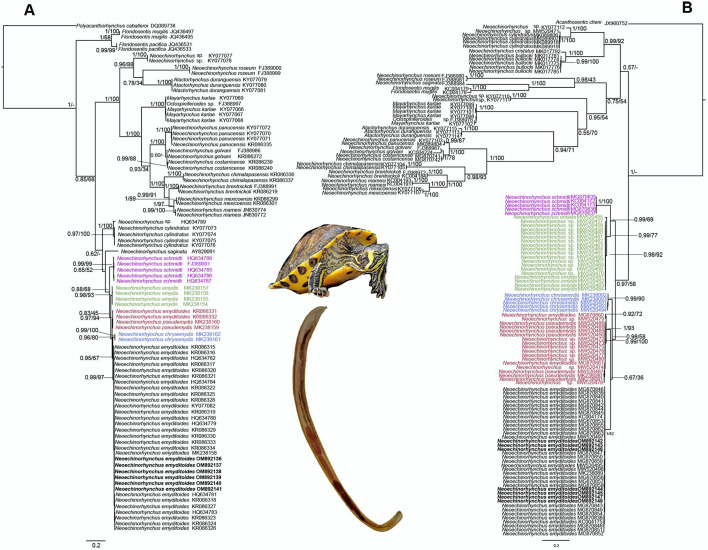
Maximum likelihood tree and consensus Bayesian Inference tree inferred with LSU dataset (A) and ITS dataset (B); numbers near internal nodes show posterior probabilities (BI) and ML bootstrap values. Sequences in bold were generated in this study.

The LSU dataset was conformed with 830 characters and 82 sequences, including 23 sequences of 10 *Neoechinorhynchus* species parasitizing teleost fishes and 46 sequences of five *Neoechinorhynchus* species associated to emydid turtles plus other sequences from diverse genera that were used as outgroups (see Table 1). The phylogenetic analyses inferred with ML and BI yielded that the genus *Neoechinorhynchus* is polyphyletic, due to the fact that several genera that were used as outgroups such as *Mayarhynchus* and *Atactorhynchus* from the Neoechinorhynchidae were nested inside of *Neoechinorhynchus* (Fig. 4A). The five species of *Neoechinorhynchus* analysed associated with emydid turtles formed a clade, which was subdivided into five subclades that correspond with each species analysed as *N. pseudemydis*, *Neoechinorhynchus chrysemydis* Cable and Hopp, 1954, *N. emydis*, *N. schmidti* and *N. emyditoides* with high bootstrap values and Bayesian posterior probabilities (Fig. 4A). In addition, all the samples (including six newly generated sequences) identified as *N. emyditoides* recovered in the four localities from northern and southeastern Mexico formed a subclade together with other sequence identified previously as *N. emyditoides* available in the GenBank (MK238158) from Oklahoma, USA (Fig. 4A). The intraspecific genetic divergence among the isolates of *N. emyditoides* was low that ranged from 0 to 1.6%. Two juvenile worms previously identified as *N. emyditoides* from Monterrey, Mexico (KR086331–332) were nested in a subclade together with other two sequences available in GenBank (MK238159–160) identified as *N. pseudemydis*, a parasite of the red-eared slider turtle from Oklahoma, USA. This subclade was supported with high bootstrap values and Bayesian posterior probabilities (Fig. 4A).

A second dataset with the ITS was conformed with 886 characters and 120 sequences. This alignment included 27 sequences of 12 *Neoechinorhynchus* species parasitizing teleost fishes and 84 sequences of five *Neoechinorhynchus* species associated with emydid turtles plus other sequences from diverse genera that were used as outgroups (see Table 1). The phylogenetic analyses inferred with ITS dataset agreed with the LSU tree with respect to polyphyly of *Neoechinorhynchus* due to the fact that the several genera that were used as outgroup taxa namely *Mayarhynchus*, *Atactorhynchus* and *Floridosentis* from the Neoechinorhynchidae were nested inside *Neoechinorhynchus* (Fig. 4B). The five species of *Neoechinorhynchus* associated with emydid turtles formed a single clade, which was subdivided into five subclades that correspond with each species analysed as *N. pseudemydis*, *N. chrysemydis*, *N. emydis*, *N. schmidti* and *N. emyditoides* with high bootstrap values and Bayesian posterior probabilities (Fig. 4B). All samples (including seven newly generated sequences) identified as *N. emyditoides* recovered in the four localities from northern and southeastern Mexico formed a subclade together, with other three sequences identified previously as *N. emyditoides* available in the GenBank dataset (MW520495–497) from Oklahoma and Texas, USA (Fig. 4B). The intraspecific genetic divergence among the isolates of *N. emyditoides* was low that ranged from 0 to 0.5%. Two juvenile worms previously identified as *N. emyditoides* from Monterrey, Mexico (MG870860–861) were nested in a subclade together with other seven sequences available in GenBank (MK238090–901, MW520486–491) identified as *N. pseudemydis*, plus other nine sequences of *Neoechinorhynchus* sp. (MW520472–480), recovered from the ostracods of the genus *Physocypra* from Oklahoma, USA (Fig. 4B).

### Haplotype network

**Fig. 5.**
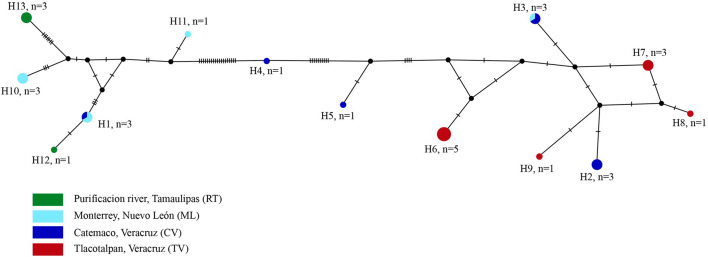
Haplotype network of samples of *Neoechinorhynchus emyditoides*, built with the gene cytochrome c oxidase subunit 1 (*cox1*) from mitochondrial DNA. Each circle represents a haplotype, with size proportional to the haplotype's frequency in the populations.

A haplotype network was built with the *cox1* alignment of 546 bp and contained sequences of 29 specimens (including three newly generated sequences) identified as *N. emyditoides* from four populations two from northern Mexico (localities; Monterrey, Nuevo León and Purificación river, Tamaulipas) and other two from southeastern Mexico (localities; Catemaco, Veracruz and Tlacotalpan, Veracruz). The haplotype network revealed the presence of 13 haplotypes, two of them (H1, H3) were shared between the localities of Catemaco, Veracruz and Monterrey, Nuevo León and 11 were unique haplotypes (Fig. 5). The level of haplotype diversity (Hd = 0.913) was very high, and nucleotide diversity was low (pi = 0.03728) among the populations (Table 3). We detected low values of *F*_st_ between the two populations from southeastern Mexico [localities; Catemaco, Veracruz and Tlacotalpan Veracruz (*F*_st_ = 0.1603)] and between the two populations from northern Mexico [localities; Monterrey, Nuevo León and Purificación river, Tamaulipas (*F*_st_ = 0.1848)] (see Table 4).

**Table 3.** Molecular diversity indices and neutrality tests calculated for *cox 1* datasets among the populations of *Neoechinorhynchus emyditoides* used in this study

Locality	*n*	*S*	*H*	Hd ± s.d.	Pi ± s.d.	*K*	Tajima's *D*	Fu's *F*s
TV	10	7	4	0.711 ± 0.117	0.00541 ± 0.00087	2.955	0.82363	1.511
CV	8	40	5	0.857 ± 0.108	0.02643 ± 0.00963	14.428	−0.34625	3.359
ML	7	41	4	0.810 ± 0.130	0.02651 ± 0.01121	14.476	−0.89590	4.484
RT	4	13	2	0.500 ± 0.265	0.01190 ± 0.00631	6.500	−0.84307	4.605
All	29	51	13	0.931 ± 0.022	0.03728 ± 0.00307	20.352	1.80641	4.715

*n*, number of sequences; *H*, number of haplotypes; *S*, number of segregating sites; Hd, haplotype diversity; Pi, nucleotide diversity; *K*, average number of nucleotide differences; TV, Tlacotalpan, Veracruz; CV, Catemaco, Veracruz; ML, Monterrey, Nuevo León; RT, Purificación river, Tamaulipas

**Table 4.** Pairwise *F*_st_ values estimated for cox (under the diagonal) and *F*_st_ *P* values (above the diagonal)

	TV	CV	ML	RP
TV	–	0.00164 ± 0.0005	0.00117 ± 0.0002	0.00557 ± 0.0009
CV	0.1603	–	0.20871 ± 0.0047	0.03185 ± 0.0019
ML	0.7522	0.4227	–	0.02864 ± 0.0015
RP	0.8925	0.6296	0.1848	–

TV, Tlacotalpan, Veracruz; CV, Catemaco, Veracruz; ML, Monterrey, Nuevo León; RT, Purificación river, Tamaulipas.

Significance level = 0.05.

## Discussion

To the best of our knowledge, two species of the genus *Neoechinorhynchus* (*N. schmidti* and *N. emyditoides*) associated with emydid turtles have been recorded in Mexico (Bravo-Hollis, 1946; García-Varela *et al*., 2011; Pinacho-Pinacho *et al*., 2018*a*, 2018*b*). The current records suggest that the species do not have a sympatric distribution; for example, *N. emyditoides* has been recorded from northern Mexico to the Papaloapan River basin, which is considered the second largest hydrobiological system in southeastern Mexico, whereas *N. schmidti* has been recorded in the Centla Swamps, which is one of the largest wetlands in southeastern Mexico and is formed by the delta of the Grijalva and Usumacinta rivers (González-Ramírez and Parés-Sierra, 2019). The species *N. emyditoides* was described in red-eared slider turtles (*T. scripta elegans*) from the St. Francis River, Arkansas, USA (Fisher, 1960). This acanthocephalan is considered one of the biogeographical core parasite fauna of emydid turtles in the eastern USA (Barger, 2004; Barger and Nickol, 2004). In the current study, adults recovered from several emydid turtles from northern and southeastern Mexico were identified as *N. emyditoides*. Our observations and morphometric data found remarkable morphological differences among the populations from Mexico (see Table 2), including the contour posterior end of the females (Fig. 3). The inclusion of molecular characteristics in this study was key to achieving a better understanding of the phenotypic plasticity of *N. emyditoides* along its distribution range. We demonstrated that the posterior shape of females exhibits great phenotypic plasticity, which is essential for delimitation of the 10 recognized species associated with freshwater turtles. Therefore, the addition of molecular data should be necessary on the description and delimitation of species of the genus *Neoechinorhynchus* associated with emydid.

The haplotype network analysis of *cox1* sequences inferred with 29 sequences revealed the presence of 13 haplotypes, two of which (H1, H3) were shared between the localities of Catemaco, Veracruz and Monterrey, Nuevo León, and 11 were unique haplotypes (Fig. 5). The two populations from southeastern Mexico (Catemaco and Tlacotalpan, Veracruz) had low *F*_st_ value (0.1603), suggesting genetic flow between both populations, which can be explained by the fact that both localities belong to the same hydrological basin (González-Ramírez and Parés-Sierra, 2019). In contrast, the two localities from northern Mexico (Monterrey, Nuevo León and Purificación river, Tamaulipas) did not belong to the same freshwater system. However, the value of *F*_st_ was also low (0.1848), which suggests that definitive or intermediate hosts have the capacity to disperse and maintain the connection between the two localities.

Our phylogenetic analyses inferred with the LSU and ITS datasets confirmed that *Neoechinorhynchus* is polyphyletic, with most of its members nested in several independent clades that did not share a common ancestor. However, a main clade was formed with the five species of *Neoechinorhynchus* associated with emydid turtles. This clade contained three species distributed in the eastern USA (*N. emydis*, *N. pseudemydis* and *N. chrysemydis*), one in southeastern Mexico (*N. schmidti*) and one species (*N. emyditoides*) distributed from the eastern USA to southeastern Mexico (Fig. 4A and B). All the samples identified as *N. emyditoides* recovered from the four locations from northern and southeastern Mexico formed a subclade together with other sequences previously identified as *N. emyditoides* available in GenBank for LSU (MK238158) and ITS (MW520495–497) from Texas and Oklahoma, USA (Fig. 4A and B). Two juvenile worms from red-eared sliders in Monterrey, Mexico (KR086331–332 and MG870860–861 for LSU and ITS, respectively) previously identified as *N. emyditoides* were nested in a subclade together with other sequences identified as *N. pseudemydis*. Koch *et al*. (2021) performed one of the most extensive studies of morphological, ecological and molecular data of the snail, ostracod and turtle hosts of *Neoechinorhynchus* species from the eastern USA. Their analysis found that the sequences from northern Mexico originally identified as *N. emyditoides* (KR086331–332 and MG870860–861) corresponded to *N. pseudemydis*. The intraspecific genetic divergence estimated on the current study among the isolates of *N. emyditoides* ranged from 0.3 to 1.6% and from 0 to 0.5% for LSU and ITS, respectively. These values of intraspecific genetic divergence are similar to those previously reported for isolates of *N. chrysemydis*, *N. schmidti* and *N. emydis*, which ranged from 0.3 to 1.6% for ITS and from 0 to 0.01% for LSU (García-Varela *et al*., 2011; Koch *et al*., 2021).

Members of the Emydidae family have been divided into two monophyletic lineages recognized as the subfamilies or complexes, Deirochelyinae and Emydinae (Gaffney and Meylan, 1988; Spinks *et al*., 2016). Deirochelyinae includes six genera broadly distributed in North America but also contains a few taxa that extend across the Greater Antilles, Mexico, Central America and South America (Parham *et al*., 2013, 2015). Four of the six genera from Deirochelyinae (*Trachemys*, *Graptemys*, *Pseudemys* and *Chrysemys*), which share a common ancestor (Thomson *et al*., 2021), have been found to harbour *Neoechinorhynchus* species (Barger and Nickol, 2004). The fossil record suggests that Deirochelyine originated in North America approximately 20.91 Ma prior to the Miocene (Spinks *et al*., 2016). Barger (2004) suggested that the eastern USA represents a biodiversity hot spot for *Neoechinorhynchus* species (and the same for emydids; see Lindeman, 2013) that infect emydid turtles. The presence of *N. emyditoides* in northern and southern Mexico could have resulted from ancestral populations of emydid turtles that inhabited the eastern USA and colonized new habitats along the coasts to the south due to the relatively warm and wet temperatures during the mid-Miocene (Spinks *et al*., 2016), which is mirrored in the diversity of both the hosts and their parasites.

## Supplementary material

For supplementary material accompanying this paper visit https://doi.org/10.1017/S003118202200049X. click here to view supplementary material
