# Supplementary material for: Phenotypic plasticity, genetic structure and systematic position of Neoechinorhynchus emyditoides Fisher, 1960 (Acanthocephala: Neoechinorhynchidae): a parasite of emydid turtles from the Nearctic and Neotropical regions
Source: Parasitology. 2022 Apr 19;149(7):991–1002. doi: 10.1017/S003118202200049X (PMC10090580; doi:10.1017/S003118202200049X)
Supplement: Supplementary file 1 [file S003118202200049Xsup001.docx]

**Supplementary Table 1**. Information of the specimens sampled in this study. Sampled localities; host name; number of host examined/infected (prevalence of infection); taxonomic name; coordinates.

| **Localities** | **Host** | **Acanthocephalan species** | **Coordinates** |
| --- | --- | --- | --- |
| Huizache, Sinaloa | *Trachemys ornata* (2/0) |  | 23º 6' 47.2" N  106º 13' 45.01" W |
| Tovara Nayarit | *Trachemys ornata* (2/0) |  | 21º 32' 24.018" N  105º 13' 15.815" W |
| Tres Palos, Guerrero | *Trachemys ornata* (2/0) |  | 16º 48' 53.478" N  99º 43' 36.022" W |
| Monterrey, Nuevo Leon | *Trachemys scripta elegans* (2/2) | *N.emyditoides* | 25º 54' 16" N  98º 52' 4.598" W |
| Río Purificacion, Tamaulipas | *Trachemys scripta venusta* (2/2) | *N.emyditoides* | 24º 5' 21" N  99º 9' 54" W |
| Tlacotalpan, Veracruz | *Trachemys scripta venusta* (9/9)  *Kinosternon flavescens* (2/0)  *Staurotypus triporcatus* (5/0) | *N.emyditoides* | 18º 36' 38" N  95º 39' 49.16" W |
| Catemaco, Veracruz | *Trachemys scripta venusta* (7/7)  *Kinosternon flavescens* (2/0)  *Staurotypus triporcatus* (2/0) | *N.emyditoides* | 18º 22' 44.89" N  95º 7' 24.639" W |
| Pantanos de centla, Tabasco | *Trachemys scripta venusta* (5/5) | *N.schmidti* | 18º 28' 18" N  92º 39' 15.001" W |
| Holka, Yucatán | *Trachemys scripta venusta* (5/0) |  | 20º 46' 7" N  88º 58' 19.99" W |
